# A Case of Lemierre-Like Syndrome in a Pediatric Patient

**DOI:** 10.7759/cureus.58192

**Published:** 2024-04-13

**Authors:** Anjali Patel, Karen Yang, Hanna S Sahhar

**Affiliations:** 1 Medicine, Edward Via College of Osteopathic Medicine, Auburn, USA; 2 Internal Medicine, Edward Via College of Osteopathic Medicine, Spartanburg, USA; 3 Pediatric Intensive Care Unit, Spartanburg Regional Healthcare System, Spartanburg, USA

**Keywords:** mrsa mediastinitis, septic thrombophlebitis, methicillin-resistant staphylococcus aureus, lemierre-like syndrome, lemierre syndrome

## Abstract

Lemierre-like syndrome is a rare, systemic sequelae following a persistent oropharyngeal infection, leading to septic thrombophlebitis of the internal jugular vein (IJV). Lemierre syndrome is caused by the obligate anaerobic organism *Fusobacterium necrophorum*, innate to the oropharyngeal tract. Lemierre-like syndrome is due to infections caused by other organisms, including methicillin-resistant *Staphylococcus aureus *(MRSA). We are reporting a case of a five-month-old male who presented with one week of fever that was not alleviated by acetaminophen, bilateral otitis media, and left-sided cervical lymphadenopathy not alleviated with medical therapy. The patient’s clinical course continued to deteriorate as he developed respiratory distress that progressed to acute respiratory failure requiring mechanical ventilation support. Extensive laboratory investigation ruled out the causes of primary and secondary immunodeficiencies. Blood cultures were positive for MRSA, and he was treated initially with vancomycin, then switched to linezolid per ENT recommendations, and ultimately needed daptomycin and ceftaroline therapy. A computed tomography (CT) scan of the neck and chest showed deep neck space infection, bilateral loculated pleural empyema, and mediastinitis. The patient required a decortication video-assisted thoracoscopic surgery (VATS), multiple drains, and a mediastinal washout to control the MRSA infection. This report emphasizes that the rapid progression and spread of septic thrombus can become detrimental to a patient’s recovery and survival; therefore, it should be recognized early and treated promptly.

## Introduction

First described in 1936 by Andre Lemierre and only having about 14 cases per million annually, Lemierre syndrome is a rare disease characterized by the septic thrombophlebitis of the internal jugular vein (IJV).

Although there are no standardized criteria for defining a case of Lemierre syndrome, the condition results from an initial oropharyngeal infection, most commonly caused by the gram-negative anaerobic pathogen *Fusobacterium necrophorum* [1]. *Fusobacterium necrophorum* is a rare causative agent of otitis and sinusitis [2]. Although it may resemble the classic picture of Lemierre syndrome, if a clinical presentation is not preceded by an oropharyngeal infection or the infectious source is an aerobic organism, the case is likely titled Lemierre-like syndrome. Unlike Lemierre syndrome, Lemierre-like syndrome is commonly caused by *Staphylococcus aureus*, especially in cases involving very young children [3].

Although there is no consensus on the differentiation, Lemierre syndrome typically manifests with symptoms localized to an oropharyngeal infection, such as sore throat, dysphagia, neck pain, and trismus that dissipates to include symptoms reflecting IJV invasion, such as tenderness and swelling along the angle of the jaw or the sternocleidomastoid muscle [1]. Consequently, Lemierre-like syndrome can also be preceded by other facial infections, including but not limited to orbital, nasal, and ear infections [4]. Aside from the causative microorganism, both Lemierre syndrome and Lemierre-like syndrome have similar clinical progression and rapid complications that can leave the patients in critical condition if not identified and addressed in a timely manner. The most common sequelae of the primary infection are septic embolism to the lungs, while other less common presentations include septic arthritis, osteomyelitis, skin lesions, and jaundice [5]. A computer tomography (CT) scan with contrast is the best imaging modality to diagnose an IJV thrombosis and can assist in bringing Lemierre and Lemierre-like syndromes into the differential [6]. With limited cases of Lemierre-like syndrome seen, we intend to describe a case in a young infant to increase awareness of this condition and the importance of prompt treatment.

## Case presentation

We present a case of a previously healthy five-month-old male who initially presented with one week of ear tugging, irritability, and a temperature of 101.4°F, not alleviated with acetaminophen. He was diagnosed with bilateral acute otitis media and discharged from the emergency department (ED) with amoxicillin. However, the parents noted new swelling in his left lower jaw, continuous fever spikes, increased fussiness, and poor oral intake and brought the patient back to the ED the following day for evaluation. Laboratory investigations at this time were notable for white blood cell count of 23.4 × 103/µL (reference range: 6.5-13.3 × 103/µL) and increased C-reactive protein (CRP) of 27.8 mg/dL (reference range: 0.0-0.6 mg/dL). Chest X-ray (CXR) was unremarkable at this time.

The patient was admitted to the pediatric ward for his left-sided cervical lymphadenopathy in the posterior submandibular triangle on the eighth day of illness. Ampicillin-sulbactam was given once, and an otolaryngologist (ENT) was consulted. ENT recommended treatment with intravenous (IV) clindamycin and IV dexamethasone and reported that imaging was not warranted. With the improvement of the patient’s inflammatory markers and decreased left submandibular lymphadenopathy, the patient was discharged home on a 10-day course of oral clindamycin.

**Figure 1:**
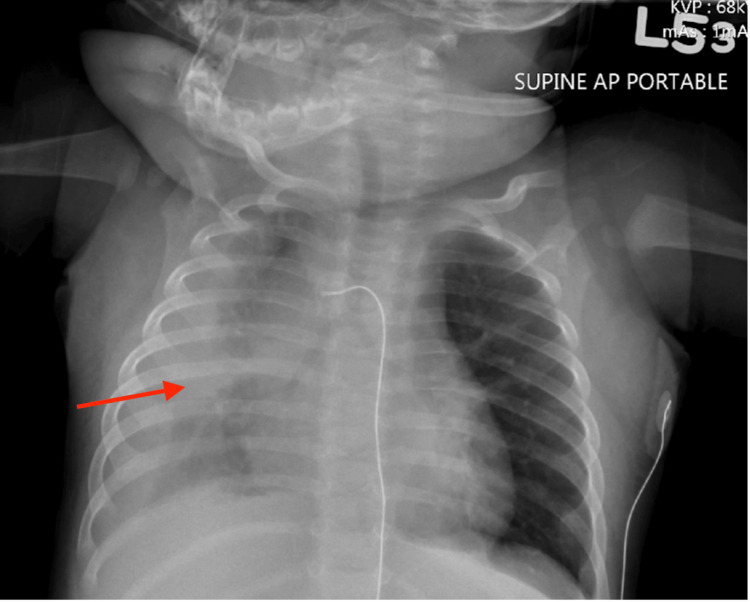
Chest X-ray with right-sided pleural effusion The initial chest X-ray of the patient when he presented with signs of respiratory distress. It shows signs of right lung infiltrate with a small, right-sided pleural effusion (arrow) AP: anteroposterior

Two days after being discharged, the patient returned for continued poor oral intake, daily fevers of 101°F, and signs of respiratory distress including grunting, nasal flaring, and retractions. He was admitted at this time and started on ceftriaxone and dexamethasone. CXR at this time showed a right lung infiltrate with a small, right-sided pleural effusion (Figure 1), and blood cultures were positive for methicillin-resistant *Staphylococcus aureus* (MRSA), for which the patient was started on vancomycin. The next day, the patient was transferred to the pediatric intensive care unit (PICU) for worsening respiratory status and required oxygenation support via a high-flow nasal cannula (HFNC). Repeat CXR showed increased right-sided opacities and pleural effusion. An echocardiogram was completed, which showed moderate pericardial effusion with normal cardiac function. On day 3 of admission, thoracentesis with chest tube drainage was performed, resulting in pleural fluid that grew MRSA. Due to suboptimal vancomycin trough levels, the patient was transitioned to linezolid for broader MRSA coverage. With non-improving respiratory conditions, the patient was intubated, and a central venous line was placed for access.

**Figure 2:**
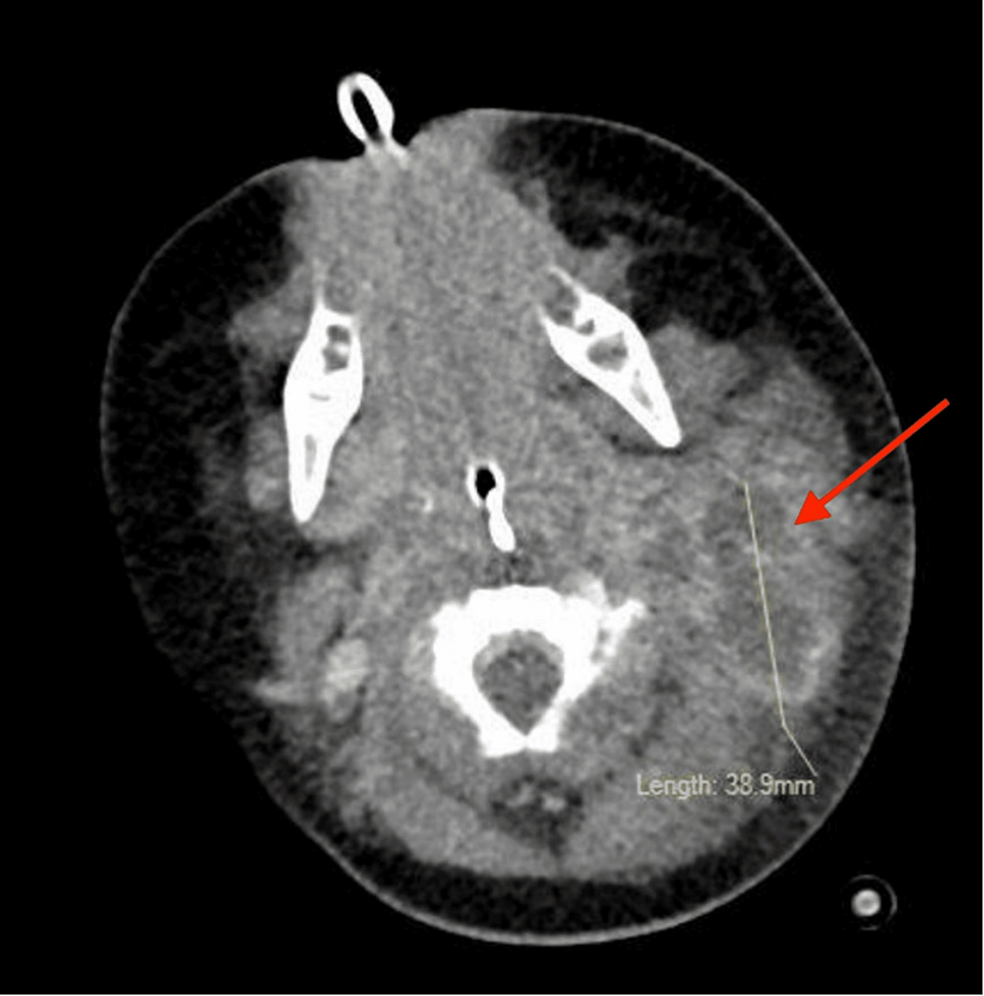
CT of the soft tissue of the neck with contrast Loculated thick-walled centrally hypodense collection within the left upper jugular chain measuring 38.9 mm that extends deep into the left sternocleidomastoid (arrow) CT: computed tomography

On day 14 of illness and day 5 of admission, a CT scan with contrast of the patient’s neck and chest showed fluid collections in the left neck with extensive involvement into the mediastinum with moderate pericardial and bilateral pleural effusions (Figure 2). The fluid collections were seen wrapped around the great vessels with complete occlusion of the left internal jugular vein and partial occlusion of the superior vena cava. Due to the evidence of deep neck space infection, the patient was transferred to a tertiary hospital for further evaluation and treatment.

On arrival at the tertiary hospital, the patient was immediately taken to the operating room for the incision and drainage of the cervical lymphadenopathy with ENT and decortication video-assisted thoracoscopic surgery (VATS) with cardiothoracic surgery, followed by the placement of a chest tube. A repeat CXR at this time showed an enlarged cardiac silhouette and right-sided pneumonia. Per recommendations from infectious disease specialists, the antibiotic was switched to daptomycin for MRSA bacteremia, and ceftaroline was added as coverage for pneumonia. The patient became hypotensive and required epinephrine infusion. A pericardial drain was placed due to the fluid collection and hypotension. The pericardial drain was removed two days later due to minimal output, and a left chest tube was placed due to increased fluid collection shown on serial CXR. The chest tube drained serous fluid. A CT angiography showed thrombi in the superior vena cava, left femoral vein, and iliac vein. Thus, a heparin infusion was started and titrated based on activated partial thromboplastin time (aPTT) to therapeutic goals before switching to enoxaparin sodium at discharge.

The patient’s blood cultures continued to be positive for MRSA bacteremia, and inflammatory markers continued to be elevated. Cardiothoracic surgery, ENT, and pediatric surgery were consulted for infection source control of the mediastinitis and recommended mediasternotomy and washout with venotomy of the inferior venous cava and repair. The patient continued his course of ceftaroline and daptomycin before transitioning to monotherapy IV ceftaroline at discharge with plans to follow up as an outpatient with infectious disease. With clinical improvement after the washout, the patient was extubated and transitioned to HFNC, followed by progressive weaning as tolerated, to room air. The patient was placed on quetiapine for two weeks prior to discharge due to having an elevated Cornell Assessment of Pediatric Delirium (CAPD) score after being off of his sedation.

Due to the patient’s suspected immunocompromised state and the severity of his infection, immunological markers were investigated. The patient had a normal total complement hemolytic activity (CH50) of 58 U/mL (reference range: 30-75 U/mL), normal dihydrorhodamine (DHR) test, elevated immunoglobulin (Ig) levels (IgA, IgE, IgG, and IgM), and deficient cluster of differentiation (CD) cell lines (CD3, CD4, CD8, CD4/CD8, CD56/CD16, and CD19) with plans for further investigation as an outpatient with the immunology team (Tables 1, 2). It is important to note that the patient was lost to follow-up due to their transfer of care to multiple higher-level medical facilities. Due to this, we are unaware if the patient was referred for further outpatient follow-up and testing, which would be necessary to confirm an underlying immunological deficiency.

**Table 1:** Cluster of differentiation cell lines A summary of the cluster of differentiation (CD) cell line markers. The percentage of all of the patient’s markers, CD3, CD4, CD8, CD56/16, and CD19, is within normal limits; however, the specific count of the markers, CD3, CD4, CD8, and CD56/CD16, and the ratio of CD4/CD8 are all deficient compared to the reference range for the patient

Cluster of Differentiation	Patient’s Values	Reference Range
CD3 count	1029 µL	2284-4776 µL
CD4 count	548 µL	1523-3472 µL
CD8 count	418 µL	524-1583 µL
CD4/CD8 ratio	1.3	1.5-3.8
CD56/CD16 count	192 µL	230-801 µL
CD19 count	330 µL	776-2238 µL
CD3 percentage	64%	52%-74%
CD4 percentage	35%	35%-53%
CD8 percentage	27%	13%-27%
CD56/CD16 percentage	12%	4%-15%
CD19 percentage	20%	17%-37%

**Table 2:** Immunoglobulin (Ig) panel results A summary of the patient’s immunoglobulin (IgA, IgE, IgG, and IgM) levels

Immunoglobulin	Patient’s Values	Reference Range
IgA	134 mg/dL	30 mg/dL
IgE	1273 IU/mL	0-60 IU/mL
IgG	1762 mg/dL	110-700 mg/dL
IgM	125 mg/dL	20-90 mg/dL

## Discussion

The patient’s initial clinical symptoms and presentation were those of acute otitis media. However, due to the quickly changing nature of the patient’s presentation and overall decline, a more severe underlying pathological process must be considered. As in the case of our patient, Lemierre and Lemierre-like syndromes classically follow head and neck infections, including otitis media and mastoiditis [5]. Lemierre-like syndrome, having a similar pathophysiology as Lemierre syndrome, is thought to begin with an initial infection that damages the mucosa at the site of infection along with nearby areas.

The principles of treatment and clinical course for Lemierre-like syndrome are similar to those of Lemierre syndrome including antibiotic therapy and consideration for surgical intervention and anticoagulation. Antibiotic therapy should be specifically tailored to bacterial culture results and susceptibilities [7,8]. If patients are persistently bacteremic or clinically worsening despite receiving the appropriate antibiotic regimen, further evaluation with CT for the extension of infection and antimicrobial susceptibility testing should be considered. Limited data is reported on the role of anticoagulation in these cases but has been reserved for those with severe progression of thrombosis or continued clinical signs of bacteremia and sepsis after receiving 5-7 days of antimicrobial treatment. Interventional procedures including drainage, debridement, and ligation/excision of the IJV are guided by clinical circumstances and only considered in severe cases [2].

It has been reported repeatedly that a persistent *S. aureus* bloodstream infection can lead to rapid spread and an increased overall infectious burden. In cases of Lemierre syndrome, *F. necrophorum* is described as releasing toxins that cause localized hypercoagulable activity leading to thrombophlebitis. Thrombophlebitis ultimately allows for the release of septic emboli into the systemic circulation targeting many organs, primarily the lungs [6]. *Staphylococcus aureus*, a common cause of gram-positive sepsis, is also known to cause thrombotic complications, both macrovascular and microvascular, along with disseminated intravascular coagulation (DIC). The currently recommended and approved antibiotics for *S. aureus* infections include vancomycin and linezolid, with the latter being seen to have a greater efficacy in MRSA infections. The early and proper administration of these antibiotics however is crucial to reducing the risk of adverse effects associated with *S. aureus* infections [9].

Of note, the patient’s immunological laboratory results show evidence of adequate innate immunity in response to the bacterial infection through the elevated immunoglobulins and a deficient adaptive immune response through the impaired CD cell markers’ counts. The CD markers are surface antigens responsible for the phenotyping of immune cells. CD3 is found on the surface of all T-cell lymphocytes and is expressed in the thymus via pro-thymocytes, where T-cells are first formed [10]. CD4 cells are known as T-helper cells, while CD8 cells are known as cytotoxic T-cells. Both CD4 and CD8 cell markers are crucial in the recognition and destruction of foreign particles [10]. CD16 and CD56 markers are both found on natural killer cells for antibody-dependent cell-mediated cytotoxicity. CD19 is one of the earliest markers found on B lymphocytes and is lost when the B-cell matures into a plasma cell [10].

The major T-cell primary immunodeficiencies include DiGeorge syndrome, also known as congenital thymic aplasia; chronic mucocutaneous candidiasis; hyper-immunoglobulin E syndrome; and Interleukin (IL) 12 receptor deficiency [11]. Patients with DiGeorge syndrome present with cardiac defects, abnormal facies, thymic hypoplasia, cleft palate, hypocalcemia secondary to parathyroid aplasia, and 22q11 microdeletion (CATCH-22). Chronic mucocutaneous candidiasis presents with persistent noninvasive *Candida albicans* infections of the skin and mucous membranes. Hyper-IgE syndrome presents with non-inflamed staphylococcal abscesses, retained baby teeth, coarse facial structures, dermatological problems such as eczema, elevated IgE, and bone fractures. IL-12 receptor deficiency presents with disseminated mycobacterial and fungal infections [11]. Interestingly, our patient’s clinical presentation does not reflect those of the primary T-cell deficiencies. This finding highlights the importance of evaluating a patient’s immune response to an infectious state, especially when the infection is recurrent or not being successfully treated with adequate antibiotic usage, such as in the case of our patient. It is likely that our patient is susceptible to more infections, which, if not able to be entirely prevented, can be expected and addressed in a quicker time frame. 

Our patient’s case was complicated by an unclear initial hospital course and delayed presentation to the ED. The lack of clinical symptoms that would increase suspicion and necessitate a more thorough workup delayed the patient’s antibiotic treatment and admission, leading to rapid decline and progression.

## Conclusions

It can be observed from this case that there are many routes from which bacteria can enter an individual’s bloodstream and seed into any part of the human body. Although rare, Lemierre-like syndrome is a complication that can have drastic outcomes and could be fatal. Our patient’s clinical course started with a typical, recurrent upper respiratory infection; however, the infection was resistant to treatment, and the patient continued to deteriorate into acute respiratory failure with signs of deep neck space infection, bilateral loculated pleural empyema, and mediastinitis requiring the VATS procedure and mediastinal washout. Our patient’s laboratory findings demonstrated a pan-immunodeficiency in multiple T lymphocyte markers, which may have been a crucial factor in the severity of the infection. It is evident from this case, along with the few other rare presentations of Lemierre-like syndromes, that patients with head and neck infections that do not appear to improve with antibiotic treatment over a significant course of time should be further evaluated for other pathological processes.
